# Automatic MRI segmentation of pectoralis major muscle using deep learning

**DOI:** 10.1038/s41598-022-09280-z

**Published:** 2022-03-29

**Authors:** Ivan Rodrigues Barros Godoy, Raian Portela Silva, Tatiane Cantarelli Rodrigues, Abdalla Youssef Skaf, Alberto de Castro Pochini, André Fukunishi Yamada

**Affiliations:** 1 Department of Radiology, Hospital Do Coração (HCor) and Teleimagem, São Paulo, SP Brazil; 2 Department of Diagnostic Imaging, Universidade Federal de São Paulo – UNIFESP, Rua Napoleão de Barros, 800, São Paulo, SP 04024-002 Brazil; 3 Data Scientist XP Inc., São Paulo, Brazil; 4 ALTA Diagnostic Center (DASA Group), São Paulo, Brazil; 5 Department of Orthopedics and Traumatology, Universidade Federal de São Paulo (UNIFESP), São Paulo, SP Brazil

**Keywords:** Computational models, Image processing, Machine learning, Anatomy, Health care, Medical research

## Abstract

To develop and validate a deep convolutional neural network (CNN) method capable of selecting the greatest Pectoralis Major Cross-Sectional Area (PMM-CSA) and automatically segmenting PMM on an axial Magnetic Resonance Imaging (MRI). We hypothesized a CNN technique can accurately perform both tasks compared with manual reference standards. Our method is based on two steps: (A) segmentation model, (B) PMM-CSA selection. In step A, we manually segmented the PMM on 134 axial T1-weighted PM MRIs. The segmentation model was trained from scratch (MONAI/Pytorch SegResNet, 4 mini-batch, 1000 epochs, dropout 0.20, Adam, learning rate 0.0005, cosine annealing, softmax). Mean-dice score determined the segmentation score on 8 internal axial T1-weighted PM MRIs. In step B, we used the OpenCV2 (version 4.5.1, https://opencv.org) framework to calculate the PMM-CSA of the model predictions and ground truth. Then, we selected the top-3 slices with the largest cross-sectional area and compared them with the ground truth. If one of the selected was in the top-3 from the ground truth, then we considered it to be a success. A top-3 accuracy evaluated this method on 8 axial T1-weighted PM MRIs internal test cases. The segmentation model (Step A) produced an accurate pectoralis muscle segmentation with a Mean Dice score of 0.94 ± 0.01. The results of Step B showed top-3 accuracy > 98% to select an appropriate axial image with the greatest PMM-CSA. Our results show an overall accurate selection of PMM-CSA and automated PM muscle segmentation using a combination of deep CNN algorithms.

## Introduction

Muscle size is a determinant of muscle strength during single-joint and multi-joint movements^1–4^. During the assessment of sports performance, power output determined by muscle strength and joint velocity are important for determining the optimal load for resistance and power training^5–9^. Magnetic resonance imaging (MRI) has been used to assess muscle size and found to significantly correlate with joint power in single-joint^10^ and multi-joint movements^1^.

Sports activities including weightlifting require powerful movement of the upper body. Bench press exercise is a multi-joint movement commonly used for improving upper body performance. The Pectoralis major muscle (PMM) and tendon (PMT) are main contributors to bench press movement, as shown in electroneuromyography studies^11,12^. Imaging evaluation of the pectoralis major is paramount when an injury is suspected. MRI is the gold standard for diagnosis of an acute pectoralis major tear useful to identify the location, size and severity of the lesion and to also for treatment planning^13^.

The PMM and tendon have a complex musculotendinous anatomy that is often misunderstood by both radiologists and surgeons non-familiar with its morphology. As a result, published descriptions of PM ruptures have been inconsistent with the actual musculotendinous morphology. Moreover, the literature lacks an injury classification system that is consistently applied and accurately reflects surgically relevant, anatomic injury patterns. These inconsistencies can affect the surgical planning for anatomic repair of the PM and make meaningful evaluation of repair techniques and treatment outcomes not optimal^14^. Therefore, there is an opportunity to evaluate the application of emerging deep learning-based MRI algorithms for complex segmentations, such as the pectoralis major muscle described in this work.

Multiple approaches have been described to segment upper extremity muscles, mostly including rotator cuff muscles using manual or semi-automated time-consuming strategies^4,15^ and recently using deep learning as an accurate method^16^. However, no prior studies have evaluated a workflow to select a specific MR image of the pectoral major muscle and to determine the PMM-CSA. The objective of our study was to develop deep convolutional neural networks (CNN) to identify an axial image slice from a routine axial T1-weighted PM MRI and another CNN to segment the PM muscle on an axial view. We hypothesized that axial slice segmentation using a SegResNet^17^ and a method using OpenCV2 (version 4.5.1, https://opencv.org) for PMM-CSA selection would achieve high accuracy as compared with a reference standard of manual axial slice image selection and manual muscle segmentation, respectively, potentially contributing to diagnostic interpretation and athlete training management.

## Materials and methods

All methods were performed in accordance with the relevant guidelines and regulations of our institution. A total of 134 MRI examinations obtained between 2004 and 2021 were collected retrospectively, regardless of indication. The PM MRIs were performed using a 1.5 T system (Siemens, Erlangen, Germany) within our institution (hereafter referred to as “internal”), using parameters and imaging planes available as outlined in Table 1. Assessments and manual segmentations for this study were performed using Osirix v6.0 (Pixmeo, Switzerland) by two experienced musculoskeletal radiologists with 12 and 10 years of experience blinded to age, gender, prior imaging report, clinical records, and group.

**Table 1:** Imaging characteristics of training dataset.

Parameter	Value
Total of MRI studies (N)	91
Number of axial images per study*	33.86 ± 5.08
Number of axial images containing Pectoralis Major Muscle per study*	29.59 ± 4.55
Image width**	320–512
Image height**	320–512

*Data are mean ± standard deviation. ** Data are minimum and maximum pixel measurements.

**Data are minimum and maximum pixel measurements.

Pectoralis major MRIs were obtained with the patient in a supine position, head first, and using a dedicated coil. The field of view was adapted to the patient’s body habitus. Only T1-weighted axial images were used for our study. This image was used as it is recognizable and provides a representative cross-section of PM muscle.

No cases had intra-articular or intravenous contrast injection. The exclusion criteria used were poor image quality, bilateral PM images in the same field of view, and different imaging acquisition protocol. A total of 91 axial T1-weighted PM MRIs met inclusion criteria. Two steps were developed for PMM-CSA selection: Step A uses a deep CNN model for axial pectoralis muscle segmentation. Step B uses OpenCV2 (version 4.5.1, https://opencv.org) for PMM-CSA selection.

### Step A (segmentation model)

**Figure 1:**
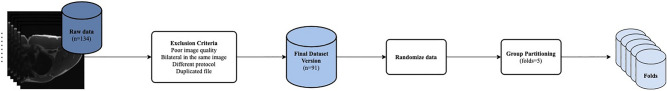
The flowchart depicts the project's exclusion criteria and the workflow for data partitioning, as well as the final research population.

For the segmentation task, we used the original U-Net^18^ as a starting point and then explored other networks: Enhanced U-Net^19^ and SegResNet^17^. We tuned the hyperparameters like dropouts, learning rate, number of groups for Group Normalization^20^, sampling size, mini-batch size, and compared the results. We then performed cross-validation using stratified group k-fold to evaluate our model. To obtain a good partition of our entire dataset, the process of partitioning satisfied several conditions: (1) data were randomized before the split, (2) split was applied to raw data, (3) leakage was avoided using group k-fold to keep all patient examples together in one set, Fig. 1.

**Figure 2:**
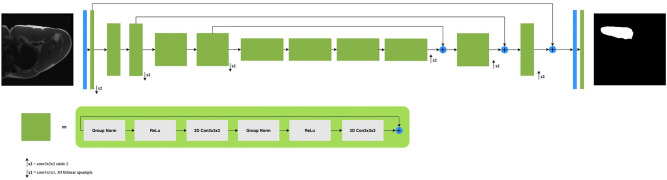
The architecture of the three-dimensional convolutional neural network (3D CNN) model used for PM segmentation. Input is a 3D axial T1-weighted PM MRI, followed by a 3 × 3 × 3 3D convolutional layer with 32 filters. Each green block is a ResNet block with Group Normalization. The output of the decoder has the same spatial size as the input, followed by a softmax function.

Our best model was a single SegResNet^17^, Fig. 2, with 2 classes (0: background, 1: pectoralis muscle) trained from scratch, which was developed by Andriy Myronenko et al.^17^. Briefly, the architecture uses an asymmetrically larger encoder to extract image features and a smaller decoder to recreate the segmentation mask in an encoder-decoder-based CNN architecture^17,21^. 3D axial T1-weighted PM MRIs were the input, followed by an initial 3 × 3 × 3 3D convolutional layer with 32 filters. The encoder part consists of four layers and uses ResNet blocks^18^. The first layer contains one ResNet block. The second and third layers contains two blocks. The last down-sampling layer comprises four blocks per spatial level. Each ResNet-like block consists of two successive Group Normalization followed by rectified linear unit (ReLU) activation function and 3 × 3 × 3 3D convolutions. A dropout layer of 0.2 was applied. The structure followed a common CNN approach where the input dimensions are progressively downsampled by a factor of two and concurrently increase the feature size by two. The decoder only contains a single block per spatial level. The up-sampling operations were performed using 1 × 1 × 1 3D convolutions to reduce the number of features and 3D bilinear upsampling to double the spatial dimension. The encoder output of the equivalent spatial level is then added. The final layer consisted of a 1 × 1 × 1 3D convolution followed by a softmax function, resulting in an output pixel-wise prediction score for each class. We did not use the variational autoencoder (VAE) branch during training. The model was trained using Python 3.8 (Python Software Foundation, Beaverton, OR) and the MONAI library (v0.6.0, https://monai.io/) with Pytorch 1.8.1 (Facebook's AI Research lab) backend. The training dataset was split using group partitioning (5-folds). For the architectures trained, we first used Medical Model ARchive (MMAR) pre-trained models provided by NVIDIA Clara Train (https://developer.nvidia.com/clara). Then, we trained the model end-to-end from scratch with all layers unfrozen. As an input, the MRIs were downsampled to 192 × 192 × 16 voxels. Data augmentation techniques were performed using MONAI transformations, such as spacing to resample the input image into a specified output voxel spacing, 3D orientation method that performs right to left on the first dimension, anterior to posterior on the second, superior to inferior on the third image, scale image intensity ranging between 0–1, spatial padding to ensure at least 192 × 192 (width x height), random affine, crop random fixed sized regions, Gaussian noise, and random flips along all spatial axis. As a normalization feature, we did not use Batch Normalization because of its intrinsic dependence on the training batch. Instead, we employed Group Normalization^20^ with a mini-batch size of 4 to attempt to attain the benefits of normalization without relying on batch statistics and, most essentially, without sacrificing performance compared to Batch Normalization. We used the Adam optimizer^22^ with an initial learning rate of 5e-4 including weight decay of 2e-5. The learning rate scheduler used was cosine annealing. As a loss function, we computed both Dice Loss and Focal Loss and returned the sum of these two losses. The model was trained for 1000 epochs with early stopping on an Ubuntu 20.04 workstation with a single NVIDIA V100 Tensor Core Graphics Processing Unit. Overall, five models were trained (one per fold) with a training time of roughly three hours per fold. To evaluate our models, we tested on internal patient scans to output predictions in 2 classes: background and pectoralis muscle. Finally, we averaged the model predictions and compared them with manual segmentations using the MONAI’s Mean Dice metric.

### Step B

**Figure 3:**
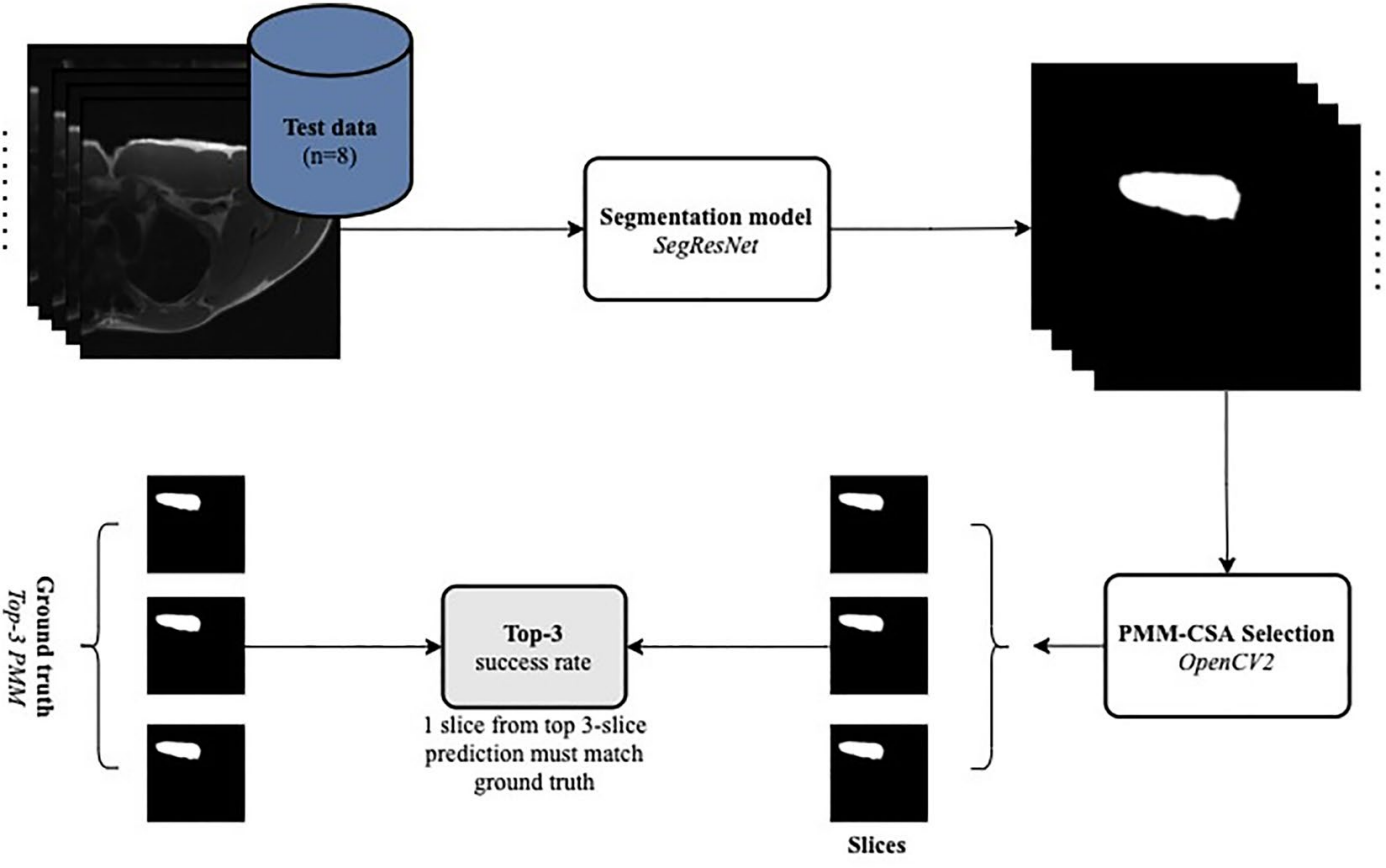
Workflow for PMM-CSA selection. The model predictions (segmentations) from the architecture trained were used for PMM-CSA selection, as well as the ground truth. The top-3 largest PMM-CSA slices were selected using OpenCV and compared with the ground truth.

For PMM-CSA selection, we first calculated the area of the pectoralis muscle present on each slice from each MRI — we performed this on both model predictions and manual segmentations from the test set. To measure the size of the pectoralis muscle region, we simply calculated, using OpenCV2 (version 4.5.1, https://opencv.org), the ratio of the pectoralis muscle area to the total area of the current slice. Then, we compared both results using the top-3 accuracy, Fig. 3.

### Statistics analysis

Descriptive statistics are reported with percentage and mean ± standard deviation (SD). For step A, the Mean Dice score was used to assess the similarity between the manual segmentations and the model predictions (segmentations). A Dice score of 1.00 is a perfect similarity. We also obtained the Hausdorff Distance (HD) score using the euclidean distance for the segmentation model tests. For step B, a top-3 success rate was used to evaluate the PMM-CSA selection performance. The top-3 success rate was determined by comparing the manually segmented ground truth to the model predictions. The prediction was considered accurate if one of the top-3 slices with the largest cross-sectional area from the segmentation model matched the top-3 slices with the largest cross-sectional from manual segmentations.

### Ethics approval

Our study was IRB-approved and complied with Health Insurance Portability and Accountability Act (HIPAA) guidelines with exemption status for individual informed consent. The research was reviewed and approved by Universidade Federal de São Paulo institutional review board and registered at the Plataforma Brasil.

## Results

### Step A

The model was trained on 91 scans from unique male patients with a mean age of 36.27 ± 8.62 years. The model was tested on 8 internal scans (all male patients, mean age, 35.92 ± 8.53 years). Imaging characteristics regarding training dataset for Step A are outlined in Table 1.

**Figure 4:**
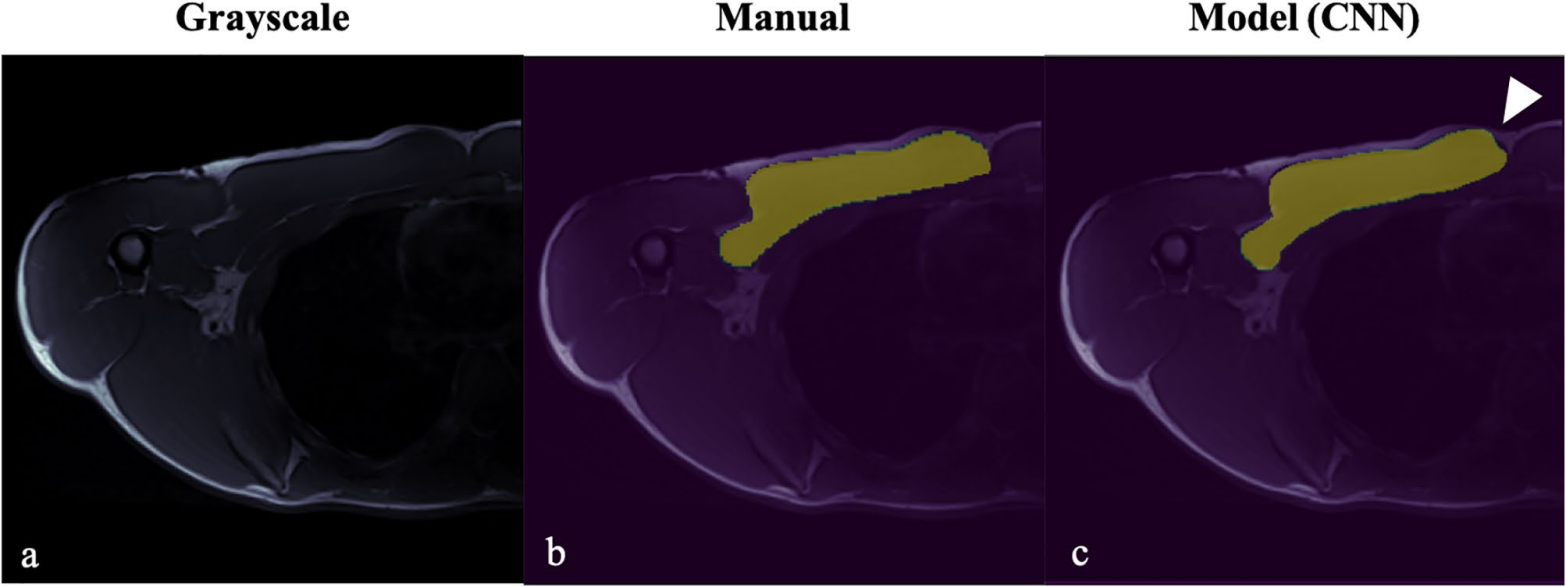
Example of accurate PM muscle segmentation using model B, with normal muscle appearance (**a**) grayscale axial T1 FSE image, (**b**) manual tracing, and (**c**) model prediction by CNN.

Manual segmentations were accomplished in approximately 15 min per scan. Training took 3 h per fold (training was run 5 times). A total of 8 scans from unique patients were collected (mean age, 35.92 ± 8.53 years) for the testing set. Overall, mean muscle segmentation Mean Dice score for internal test dataset was 0.94 ± 0.01 and are outlined in Table 2. An example of accurate muscle segmentations from the model trained is illustrated in Fig. 4.

**Table 2:** Mean-Dice and Hausdorff scores for Pectoralis Major muscle segmentation.

Total (N = 8)	Background	Pectoralis Major	Mean	Standard Deviation
Test Case 1	*0.99/**13.34	*0.92/**13.34	*0.96/**13.34	*0.04/**0.00
Test Case 2	*0.99/**6.32	*0.93/**7.34	*0.96/**6.83	*0.04/**0.72
Test Case 3	*0.99/**7.68	*0.94/**7.68	*0.97/**7.68	*0.03/**0.00
Test Case 4	*0.99/**13.96	*0.94/**13.96	*0.97/**13.96	*0.03/**0.00
Test Case 5	*0.99/**11.18	*0.92/**11.09	*0.96/**11.13	*0.04/**0.06
Test Case 6	*0.99/**8.77	*0.93/**8.54	*0.96/**8.65	*0.04/**0.16
Test Case 7	*0.99/**8.06	*0.95/**8.06	*0.97/**8.06	*0.02/**0.00
Test Case 8	*0.99/**21.00	*0.94/**26.47	*0.97/**23.73	*0.03/**3.87

*Mean-Dice Scores/**Hausdorff Score.

**Figure 5:**
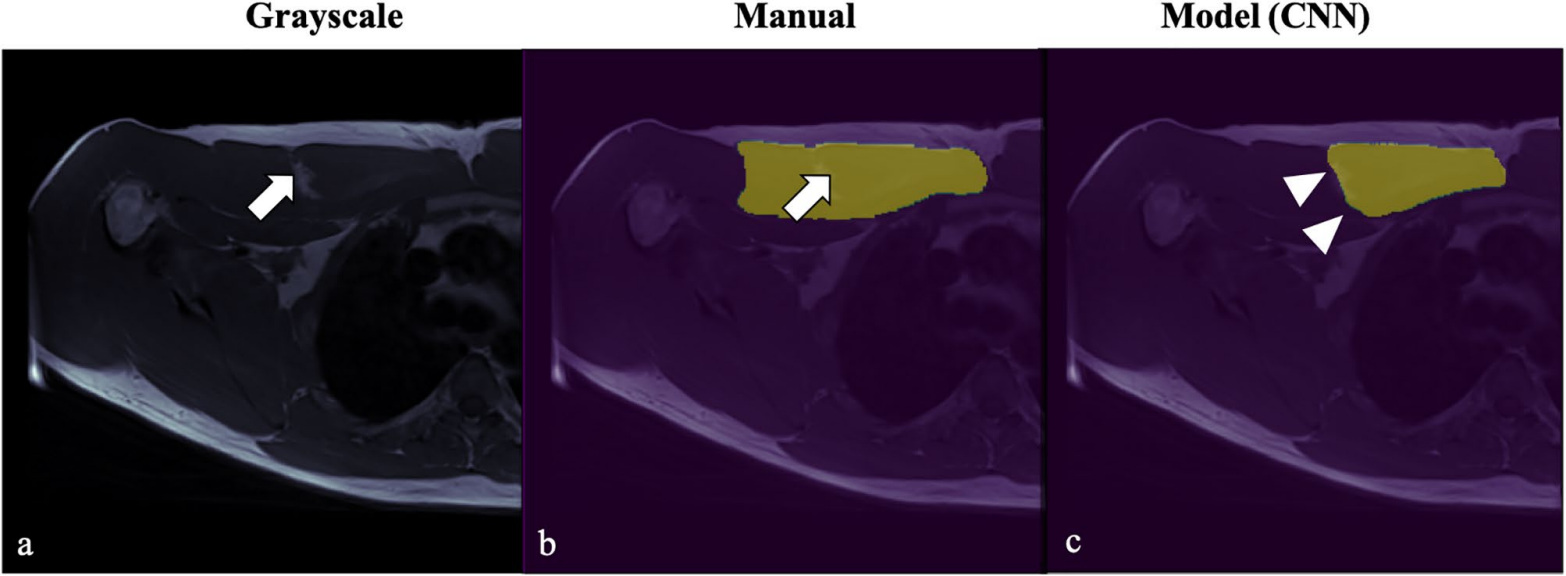
Prediction error on test images. Segmentation error at the lateral contour of the PM muscle (white arrow), due to focal fatty atrophy at the center of the muscle belly. (**a**) Grayscale Axial T1 FSE image, (**b**) manual tracing, and (**c**) CNN model prediction with underestimation of the PM muscle segmentation (arrowhead).

Although overall Mean Dice was high on internal test datasets, minor prediction errors were seen especially along the posterior contour of the pectoralis major, where the proximity with the pectoralis minor muscle was a challenging boundary to delineate. Only once, out of a total of eight test cases, did the model misclassify a larger muscle area (Fig. 5). Each automated segmentation took roughly 4 s per test scan on our workstation.

### Step B

Mean top-3 success rate to select a proper largest PMM-CSA was 100.0% (internal test dataset). Mean top-1 success rates to detect the singular ground truth Y-view were 50.0% (internal). On our workstation, selecting the largest PMM-CSA took a total time of 7.99 microseconds per test scan (each scan comprises a full T1 axial series). Finally, we performed external validation with 5 pectoralis major MR cases, not used in the previous steps, using the same acquisition protocol with a Mean-dice score of 0.91.

The pseudo code of the algorithm to detect the largest PMM-CSA is detailed as followed:
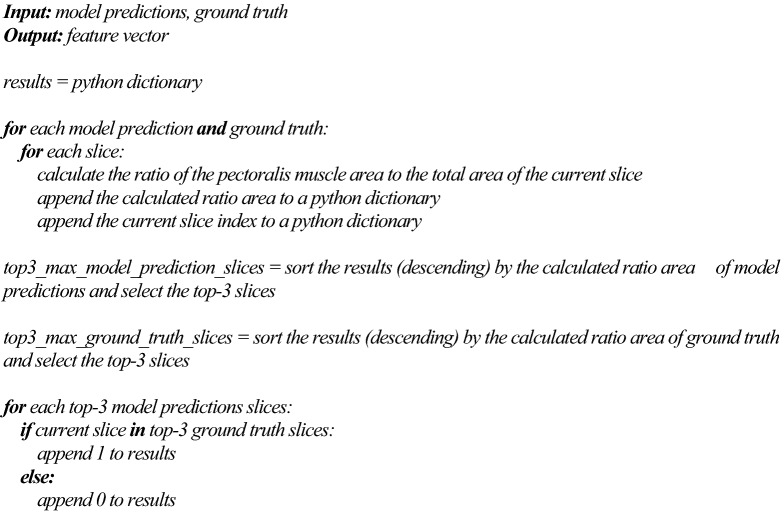


## Related work

Recent publications described newly applications for artificial intelligence such as to secure user data associated with transportation, healthcare, business^23–26^ and social activities in the context of a smart city industrial environment^27^, Greenhouse climate prediction using a Long Short-Term Memory-based Model^28^ or even using artificial intelligence to evaluate user’s next point of interest and healthcare predictions based on gated recurrent unit models^29,30^.

Deep learning techniques for musculoskeletal radiology are recently emerging and offering many new possibilities. Current literature demonstrated the application of deep learning-based MRI algorithms for ligament tear detection^31,32^, meniscus tears^33^ and rotator cuff disorders^34^ and often comparing diagnostic performance with human readers. Relevant publications comparable to this work is outlined in Table 3. Our work differs from previous studies as it is the first to evaluate a large muscle structure as the pectoralis major, using MRI containing a variety of pectoralis muscle conditions.

**Table 3:** Recent studies with deep learning-based MRI for musculoskeletal radiology.

Study	Parameter evaluated
Bien et al.^31^	Knee MRI
Germann et al.^32^	Anterior cruciate ligament tear
Fritz et al.^33^	Meniscus tear
Medina et al.^34^	Rotator cuff muscles

## Discussion

The main findings of our study are: (1) the SegResNet CNN architecture is able to accurately segment the pectoralis muscle and (2) using OpenCV2, we were able to accurately measure the size of each pectoralis muscle and select an appropriate axial T1 image containing the greatest CSA of the PM. Importantly, our results show the feasibility of these methods in a cohort of randomly selected PM MRIs.

Pectoralis major muscle size is a major determinant of bench press and throw performance^1,2,35^. Greater muscle size leads to heavier bodyweight. This can be a negative factor for certain sports, such as track and field and endurance sports^1^. However, previous studies reported that a greater muscle size reflects the power output developed by multi-joint movements, especially in weight-lifting sports (pectoralis size). Therefore, the information of the PMM size obtained in MRI studies has a significant positive impact on sports training and training programs. This study demonstrated that manual segmentation of the pectoralis major muscle is a time-consuming task, involving approximately 20 min for each case, and is usually not routinely performed during evaluation of PM MRI studies. The authors propose automated segmentation to optimize the evaluation of PMM and promote an agile evaluation of muscle size. Such evaluation ought to improve weight-lifting training, planning, and follow-up, as well as treatment planning after an eventual muscle, tendon, or myotendinous unit lesion. We focused on the axial images for their good representation of PM muscle status, and frequent use in PM injury studies^13,36,37^. Automatic slice selection methods have been previously described to identify anatomical landmarks using atlas-based approaches and deep learning^38,39^. For musculoskeletal applications, Zhou et al.^40^ had success using a CNN to select a knee sagittal slice for anterior cruciate ligament tear classification with an accuracy of 0.98. Previous study presented an accurate method for Y-view selection that can be the initial step in a workflow for automated rotator cuff muscle segmentation in shoulder MRIs^16^ with mean Dice scores of 0.94. Our automated segmentation of RC muscles showed an accuracy comparable to other deep learning methodologies for muscle segmentation.

In our study, both models were trained and tested on datasets containing a variety of PM muscle conditions (i.e., normal, MTJ tears, and PMT tears). Although our accuracy and short analysis time per image for model B are promising, areas of over-and underestimation were seen. Minor errors occurred most at muscle boundaries with adjacent fat planes and likely represent low-impact quantitative issues. More prominent errors were noted along the posterior contour of the pectoralis major close to the pectoralis minor muscle (Fig. 5).

Despite high overall and per-muscle Dice scores, strategies to improve these errors should include expanding training datasets with more cases containing confounding features in those areas. The inclusion of a larger variety of pectoralis major states may also benefit segmentation performance. Kim et al.^41^, described a potential explanation for lower supraspinatus muscle Dice score is due to variations in the cross-sectional area caused by supraspinatus tendon tears and atrophy in shoulder MRIs.

Strengths of our study include successful slice selection using a classification algorithm and demonstration of accurate automated PM muscle segmentation on routine axial T1 MRIs from a varied cohort. This approach has not been previously described for pectoralis major muscle and demonstrated robust results. Importantly, both our models were tested on datasets from studies obtained outside our institution, rendering comparable accuracies. The size of our training and testing dataset is another advantage as compared with prior studies^41,42^.

Our algorithm was not designed to quantify the degree of atrophy in each muscle or to identify PM lesions, which will require an additional stage of thresholding muscle vs. fat pixels within each segmentation. This desirable feature will be the subject of future development, which, however, depends first on robust and reliable localization of muscle boundaries, which was the focus of this study. Our manual tracing also included fatty septae and fat replacement within the boundaries of each cross-sectional area, with the future expectation of separating muscle from fat pixels using dedicated methods. Overall, such developments may allow for fast determination of pectoralis major muscle cross-sectional area in clinical workstations, which could automatically provide overlays on specific images and data on dictation platforms.

Limitations of our study include the segmentation model being trained on a single standardized axial image including PMM-CSA of a cohort of male patients. Volumetric (3D) muscle quantification using a CNN approach has been demonstrated in prior studies^42^. The use of 3D measures of muscle volume produces more accurate measures, which however requires multiple slice segmentation and longer imaging time to cover the entire shoulder and pectoralis major myotendinous unit, which is rarely accomplished in clinical practice^43^. Further studies that include PM MRI scans from female patients and other institutions are warranted for external validation of the described workflow.

## Conclusions

We demonstrate novel and accurate methods to select an axial image and segment the pectoralis major muscle using CNN architectures. Our work is the first to examine a large muscle structure and diverse cohort of patients. By offering automated and reliable muscle area quantification, our methods have potential use in training planning, lesion outcomes research, and clinical assessment of pectoralis major pathology.

## Data Availability

Available upon reasonable request from a bona fide researcher.

## Code availability

Available upon reasonable request from a bona fide researcher.
